# Supplementary material for: Epidemic spreading under mutually independent intra- and inter-host pathogen evolution
Source: Nat Commun. 2022 Oct 20;13:6218. doi: 10.1038/s41467-022-34027-9 (PMC9584276; doi:10.1038/s41467-022-34027-9)
Supplement: Supplementary file 1 — Supplementary information [file 41467_2022_34027_MOESM1_ESM.pdf]

Epidemic spreading under mutually independent intra- and inter-host pathogen evolution

Supplementary information

September 30, 2022

Contents

1 Phase diagram **1**

1.1 The lower boundary . . . 1

1.2 The upper boundary . . . 3

1.3 The case of α-mutations . . . 5

2 Evolving pathogens under SIS dynamics **6**

3 Evolving infection rate **7**

4 Correlated inter/intra-host fitness **8**

5 Numerical simulations and data analysis **10**

5.1 Simulating SIR dynamics . . . 10

5.2 Extracting σ . . . 10

5.3 Simulating SARS-CoV-2 transmission . . . 12

1 Phase diagram

Our analysis derives the σ, R_0 phase-diagram, indicating two critical transitions: the first marks the phase-boundary between the infection-free and the mutation driven states (*Lower boundary*); the second is the transition to the volatile phase, where mutations, again, are insufficient to create a sustainable breakthrough mutation (*Upper boundary*). Below we show how to analytically derive both phase-boundaries.

1.1 The lower boundary

To approximate the critical σ_c for the emergence of the mutation-driven phase, let us first consider the classic SIR model without mutations, for which $\eta(t)$ follows

$$\frac{\mathrm{d}\eta}{\mathrm{d}t} = -\alpha_0 \eta(t) + \beta_0 \overline{k} \eta(t) \big(1 - \eta(t)\big). \tag{1.1}$$

Linearizing around $\eta = 0$, one obtains

$$\eta(t) = \eta(0) e^{at}, \tag{1.2}$$

where $a = \beta_0 \overline{k} - \alpha_0 = \alpha_0(R_0 - 1)$. Therefore, in case $R_0 < 1$, we observe the well-known exponential decay back towards the infection-free state $\eta(t \to \infty) = 0$. Considering a fixed population of size of N, we express the number of infected individuals at time t by $\mathcal{I}(t) = N\eta(t)$, providing

$$\mathcal{I}(t) = \mathcal{I}_0 e^{at}, \tag{1.3}$$

where $\mathcal{I}_0 = \mathcal{I}(0)$ is the size of the initial infected population.

Next, let us add the effect of mutation. For simplicity let us first focus specifically on β-mutations, in which the infection rate evolves via $\beta_\mu = \psi_\mu \beta_0$, but the recovery rate remains fixed at α_0. This simplifies the calculation below from a bi-variate fitness parameter $\psi_{\mu,\alpha}, \psi_{\mu,\beta}$, capturing the evolution of the two rate constants, to just the one-dimensional fitness ψ_μ. Starting from a uniform population in which all pathogens have fitness $\psi_0 = 1$, we allow the pathogens to undergo the intra-host evolution. The result, as we show in the main text is that the observed inter-host fitness performs a Gaussian random walk in ψ-space. This leads to a normally distributed fitness ψ whose variance grows linearly in time as $\sigma^2 t$. Therefore, the fraction of the transmitted pathogen population whose fitness at time t is within a small margin around ψ, *i.e.* $\psi(t) \in (\psi, \psi + \mathrm{d}\psi)$, is given by the density function

$$f_t(\psi)\,\mathrm{d}\psi = \frac{1}{\sqrt{2\pi\sigma^2 t}} e^{-\frac{(\psi-1)^2}{2\sigma^2 t}}\,\mathrm{d}\psi. \tag{1.4}$$

Using this density function we express the sub-population of individuals infected by a pathogen with fitness $\psi(i, 0) \in (\psi, \psi + \mathrm{d}\psi)$ at time t by $\mathcal{I}(\psi, t)\,\mathrm{d}\psi = \mathcal{I}_0 f_t(\psi)\,\mathrm{d}\psi$. This sub-population, infected by pathogens with fitness around ψ, has $R = \psi R_0$, and therefore, using Eq. (1.3), evolves with time via

$$\mathcal{I}(\psi,t)\,\mathrm{d}\psi = \mathcal{I}_0 f_t(\psi) e^{\alpha_0(\psi R_0 - 1)t}\,\mathrm{d}\psi. \tag{1.5}$$

Equation (1.5) approximates the temporal dynamics of the ψ-fitness sub-population as an independent product of the initial infected population size, the fraction of that population having fitness ψ at time t, and the relevant fitness dependent exponential growth/decay rate. It is based on an approximation, which separates the effect of the random mutations, as captured by the Gaussian form of $f_t(\psi)$, and the natural selection, mediated by the ψ-dependent exponential growth.

Integrating (1.5) over all ψ, one obtains the dynamics of the entire infected population, providing

$$\mathcal{I}(t) = \int_\psi \mathcal{I}_0 \frac{1}{\sqrt{2\pi\sigma^2 t}} e^{-\frac{(\psi-1)^2}{2\sigma^2 t} + \alpha_0(\psi R_0 - 1)t}\,\mathrm{d}\psi. \tag{1.6}$$

Extracting all terms independent of ψ outside of the integral we obtain

$$\mathcal{I}(t) = \mathcal{I}_0 e^{\xi(t)} \int_\psi \frac{1}{\sqrt{2\pi\sigma^2 t}} e^{-\frac{(\psi-(1+\sigma^2\alpha_0 R_0 t^2))^2}{2\sigma^2 t}}\,\mathrm{d}\psi, \tag{1.7}$$

where

$$\xi(t) = \alpha_0(R_0 - 1)t + \frac{1}{2}\sigma^2\alpha^2 R_0^2 t^3. \tag{1.8}$$

In (1.7), the integral in the r.h.s. is that of a shifted Gaussian distribution, and hence it sums to unity when integrated over all ψ. Consequently, we arrive at

$$\mathcal{I}(t) = \mathcal{I}_0 e^{\xi(t)}, \tag{1.9}$$

recovering Eq. (12) of the main text (here expressed in absolute terms $\mathcal{I}(t)$ rather than relative terms $\eta(t)$).

In case $\sigma = 0$, we have no mutations, $\xi(t) = \alpha_0(R_0 - 1)t$ in (1.8), and Eq. (1.9) retrieves the solution of classic SIR model, as appears in Eq. (1.3). If, however, $\sigma > 0$, then for sufficiently large t, the cubic term in (1.8) begins to dominate and we observe an exponential proliferation in $\mathcal{I}(t)$. Most crucially, such proliferation is observed even if $R_0 < 1$. Indeed, under $R_0 < 1$, for small t, $\mathcal{I}(t)$ undergoes an exponential decay, driven by the linear term in (1.8). Yet this initial decay is followed by a guaranteed exponential growth, governed by t^3, which captures precisely the effect of the critical mutation. Of course, such super-exponential growth is rapidly inhibited as the population exhausts its pool of susceptible individuals, an effect that the linearized solution of (1.2) ignores. Still our small t approximation, leading to (1.9), is sufficient to capture the turnover effect driven by the critical mutation, even if not the ensuing dynamics that follow.

To obtain the critical time t_c for this transition we seek the minimum of $\xi(t)$, depicting the point in which the exponential curve changes its decaying trend. Hence we write $\dot{\xi}(t) = 0$, providing

$$\alpha_0(R_0 - 1) + \frac{3}{2}\sigma^2\alpha_0^2 R_0^2 t_c^2 = 0, \tag{1.10}$$

from which we obtain

$$t_c = \sqrt{\frac{2}{3\alpha_0\sigma^2}\left(\frac{1 - R_0}{R_0^2}\right)}. \tag{1.11}$$

As expected t_c is defined only for $R_0 < 1$, as, indeed, $R_0 > 1$ describes a pandemic state from the *get-go*, lacking a critical mutation.

Our solution up to this point predicts a *guaranteed* mutation transition, followed by an exponential proliferation for $t > t_c$, as, indeed, $\xi(t)$ inevitably becomes positive as $t \to \infty$. This, however, is only in the continuum limit, *i.e.* $\mathcal{I}_0 \to \infty$. For a finite initial infection, the infected population may reach $\mathcal{I}(t) = 0$ at $t < t_c$, in which case the critical mutation arrives too late, and the pathogen becomes extinct before ever reaching critical fitness.

Using the discrete nature of $\mathcal{I}(t)$ we can distinguish between two limiting cases: in case $\mathcal{I}(t = t_c) \gg 1$, we expect with high probability that the critical mutation will occur at $t \approx t_c$, giving rise to the mutation-driven phase. Conversely, if $\mathcal{I}(t = t_c) \ll 1$, the chances for such mutation vanish, and the pathogen will almost certainly become extinct before reaching critical fitness. In between these two extremes we predict a critical point when

$$\mathcal{I}(t = t_c) \sim 1, \tag{1.12}$$

in which there exists a finite probability to observe either a mutation-driven contagion or a infection-free state. Using Eq. (1.9), and taking t_c from (1.11), this translates to

$$\sigma_c \propto \frac{1}{2}\left(\frac{R_0 \ln \mathcal{I}_0}{\alpha_0^{\frac{1}{2}}(1 - R_0)^{\frac{3}{2}}} + \sqrt{\frac{2}{3}}\right)^{-1}, \tag{1.13}$$

providing the critical mutation rate σ_c for the emergence of the mutation-driven phase. Taking $\mathcal{I}_0 \gg 1$, σ_c can be further simplified to

$$\sigma_c \propto \frac{\sqrt{\alpha_0(1 - R_0)^3}}{2R_0}\frac{1}{\ln \mathcal{I}_0}, \tag{1.14}$$

recovering Eq. (15) of the main text. As explained there, we use the $\propto$ sign to note that σ_c is *of the order of* the expression on the r.h.s., but may not be exactly *equal* to it. This is because the condition in (1.12) is not that $\mathcal{I}$ is strictly equal to one, but rather that it is of order unity (~ 1).

1.2 The upper boundary

The volatile phase marks the upper boundary on σ, beyond which mutations are too rapid, and the natural selection fails to lock-in the sporadically appearing fitness gains. In this

regime the pathogen quickly reaches critical fitness, but fails to produce enough offspring that are equally fit, due to its inconsistent replication fidelity. We therefore compare the two relevant time scales: τ_1 - the time for a high fitness pathogen to sufficiently reproduce, vs. τ_2 - the time for mutations to effectively eliminate the fitness gain and drive the pathogen from $\sim \psi_{max}$, the optimal fitness, to below ψ_c.

We first begin with τ_2. To evaluate this time-scale we use the Gaussian form of $f_t(\psi)$ in (1.4) to track a *typical* pathogen, whose walk through fitness space begins at ψ_{max} and deviates from this initial fitness at a rate $\sigma\sqrt{t}$. This provides us with an approximation of the pathogen's time-evolving fitness as

$$\psi(t) = \psi_{max} - \sigma\sqrt{t}. \tag{1.15}$$

Such pathogen will lose its fitness, *i.e.* reach below ψ_c at a typical timescale of

$$\psi_{max} - \sigma\sqrt{\tau_2} = \psi_c = \frac{1}{R_0}, \tag{1.16}$$

providing

$$\tau_2 = \left(\frac{\psi_{max}R_0 - 1}{\sigma R_0}\right)^2. \tag{1.17}$$

This is the typical time by which a highly fit pathogen ($\sim \psi_{max}$) loses its fitness due to random mutations. It therefore captures the *fitness memory time*. Natural selection must act faster than this to counter this random fitness loss, and hence we now turn to calculate τ_1, capturing precisely the selection timescale.

To obtain τ_1 we use (1.3) to track the reproduction of the pathogen as

$$\mathcal{I}(t) \sim e^{\alpha_0(\overline{\psi}R_0 - 1)t}, \tag{1.18}$$

where we used the fact that the fitness changes rapidly to substitute $\psi(t)$ by its time-averaged $\overline{\psi}$. This provides with

$$\tau_1 = \frac{1}{\alpha_0(\overline{\psi}R_0 - 1)} \tag{1.19}$$

the typical time scale required for $\mathcal{I}(t)$ to observe significant growth, and hence the time window needed for the natural selection to favor the fitter pathogens. To evaluate $\overline{\psi}$ we consider the fact that Eq. (1.18) undergoes exponential growth only as long as $\psi(t) > \psi_c$, namely as long as $t < \tau_2$. We therefore seek $\overline{\psi}$ within the time window $0 \le t \le \tau_2$, providing

$$\overline{\psi} = \frac{1}{\tau_2}\int_0^{\tau_2} (\psi_{max} - \sigma\sqrt{t})\,\mathrm{d}t = \psi_{max} - \frac{2}{3}\sigma\sqrt{\tau_2}. \tag{1.20}$$

Taking τ_2 from (1.17), we substitute (1.20) into (1.19) to reach

$$\tau_1 = \frac{3}{\alpha_0(\psi_{max}R_0 - 1)}. \tag{1.21}$$

In case $\tau_2 \gg \tau_1$ the fitter pathogens have much time to proliferate (τ_1) before losing their fitness (τ_2), leading to successful spread. If, however, $\tau_2 \ll \tau_1$ the pathogens' fitness declines before it has time to sufficiently reproduce and hence its volatility bars it from spreading efficiently. The critical transition between these two states is therefore when τ_1 is of the same scale as τ_2, *i.e.*

$$\frac{3}{\alpha_0(\psi_{max}R_0 - 1)} \sim \left(\frac{\psi_{max}R_0 - 1}{\sigma R_0}\right)^2, \tag{1.22}$$

which, by extracting σ, provides

$$\sigma_c \propto \sqrt{\frac{\alpha_0}{3}} \frac{(\psi_{max}R_0 - 1)^{\frac{3}{2}}}{R_0}, \tag{1.23}$$

retrieving Eq. (16) of the main text.

1.3 The case of α-mutations

Our derivation above focused on β-mutations, setting α fixed at α_0. To complete our analysis we now allow the recovery rate α to evolve via $\alpha_\mu = \alpha_0/\psi_\mu$, and set $\beta = \beta_0$. We therefore rewrite Eq. (1.5) as

$$\mathcal{I}(\psi, t)\,\mathrm{d}\psi = \mathcal{I}_0 f_t(\psi) e^{\frac{\alpha_0}{\psi}(\psi R_0 - 1)t}\,\mathrm{d}\psi, \tag{1.24}$$

and subsequently, Eq. (1.6) as

$$\mathcal{I}(t) = \int_\psi \mathcal{I}_0 \frac{1}{\sqrt{2\pi\sigma^2 t}} e^{-\frac{(\psi-1)^2}{2\sigma^2 t} + \frac{\alpha_0}{\psi}(\psi R_0 - 1)t}\,\mathrm{d}\psi. \tag{1.25}$$

As opposed to (1.6), the integral in (1.25) cannot be solved analytically, and hence we are unable to write a closed form solution for $\mathcal{I}(t)$ as we do in (1.9). Still we can solve the α-mutation integral in (1.25) numerically and extract $\mathcal{I}(t)$ from that. With the numerical $\mathcal{I}(t)$ at hand we employ the same strategy as in the above derivation and seek the conditions that the critical mutation occurred before $\mathcal{I}(t = t_c) \sim 1$. This numerical solution yields both the lower and upper boundaries of the σ, R_0 phase-diagram, as shown in Supplementary Fig. 1 (black solid line). The analytical solutions of (1.14) and (1.23), obtained under β-mutations, are also shown (red, orange), indicating that, indeed, both types of mutations lead to the same phase boundaries (upto a multiplicative factor, see figure caption).

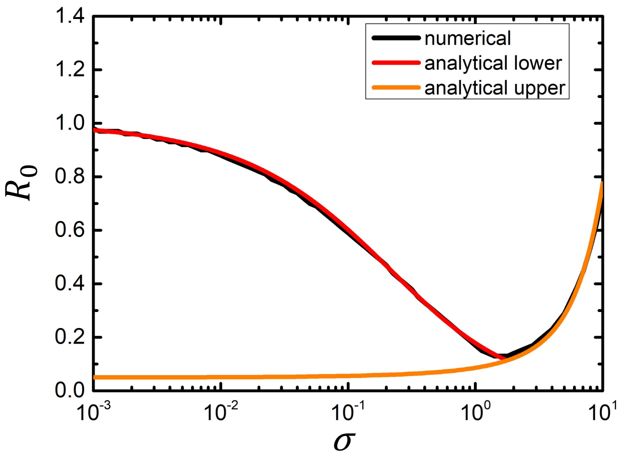

Supplementary Figure 1: **α vs. β-mutations**. To obtain the boundaries of the mutation-driven phase under α-mutations we numerically solved Eq. 1.24 and extracted the conditions under which critical mutation supersedes the pathogen extinction (black solid line). The resulting phase boundaries are in agreement with the analytical predictions of Eqs. 1.14 and 1.23, obtained under β-mutations. Note that predictions 1.14 and 1.23 are accurate up to a multiplicative factor of order unity (our transition condition is $\mathcal{I}(t = t_c) \sim 1$, not $\mathcal{I}(t = t_c) = 1$). Here, the red and orange curves were rescaled by a factor of 7 and 1.3, respectively. These results were obtained for $\psi_{\text{max}} = 20$.

2 Evolving pathogens under SIS dynamics

To complete our analysis of the SIR dynamics, we now examine the pandemic phase-diagram under the susceptible-infected-susceptible (SIS) epidemic model[1]. As expected, we find in Supplementary Fig. 2 that all results observed under SIR continue to hold also in the case of SIS. This is, of course, not surprising, as - indeed - the early stages of both epidemic models incorporate an exponential solution of the form (1.2), and hence our mathematical analysis can be readily generalized to SIS, or, for that matter, to almost all other relevant compartmental epidemic models.

Hysteresis. There is, however, an interesting phenomenon, unique to SIS that cannot be observed under SIR. As Eq. (1.14) indicates, the phase boundary between the mutation-driven and infection-free states depends on the initial condition $\mathcal{I}_0$, a dependence clearly observed in Supplementary Fig. 2e (grey solid lines). This indicates that the transition behaves differently if we approach it from the pandemic state (large $\mathcal{I}_0$) or from the infection-free state (small $\mathcal{I}_0$). To observe this we fixed the mutation rate at $\sigma = 0.1$ and gradually increased R_0, seeking the critical point where the system shifts to the mutation-driven phase. This is mapped to a vertical trajectory in the σ, R_0 plane (Supplementary Fig. 2e, yellow dashed line). At each value of R_0 we instigate an outbreak with $\eta(0) = 0.02$, and observe its long-term prevalence η_∞. For small R_0 this outbreak decays and the system reverts to the infection-free state $\eta_\infty = 0$. However, as we transition into the mutation-driven phase, here predicted at $R_0 = R_{\text{High}} \approx 0.6$, the pathogen turns pandemic and its prevalence abruptly changes to $\eta_\infty \approx 0.85$ (Supplementary Fig. 2f).

To reverse this transition the naïve approach is to push R_0 back down, to slightly below the observed critical point, for instance, by practicing social distancing to reduce transmission.

The challenge is that now, moving in the opposite direction - from large R_0 to small R_0 - our initial condition is pandemic, with prevalence of order unity ($\sim 85\%$), and hence $\mathcal{I} \sim N$. Under these conditions, Eq. (1.14) predicts that the critical R_0 is now lower than the one observed earlier, now found to be at $R_{\text{Low}} = 0.35$. This results in a hysteresis phenomenon, in which criticality occurs at different points depending on the state from which we approach the transition. Such form of direction-dependent transition is unobserved in the classic SIS dynamics, yet congruent with with other models[2–10] that incorporate feedback between a pathogen's prevalence and its capacity to spread. Here, our observed hysteresis is unique to the SIS spreading dynamics, since under SIR the long-term state is always $\eta_\infty \to 0$.

To construct the hysteretic phase-transition of Supplementary Fig. 2 we must change R_0 gradually. This ensures the system reached its η_∞ fixed-point at every instance of R_0 update. If one changes R_0 rapidly, reaching the lower bound before the system ever has time to accumulate significant prevalence, the memory effect, which depends on the direction up/down the R_0 axis may become weaker or lost.

3 Evolving infection rate

The SIR-based simulations in the main text assumed that the pathogen evolution impacted the recovery rate α, setting the infection rate β constant. The rationale is that the reproduction rate $R_i(t)$ is governed by the quotient β/α, and hence the specific value of these two parameters is unimportant, only their ratio. To complete our analysis, we now run our simulations once again, this time fixing α and allowing β to undergo mutation - with higher β, corresponding to greater fitness ψ via

$$R_i(t) = \frac{\overline{k}\beta_i(t)}{\alpha} = R_0\psi_i(t), \tag{3.1}$$

a là Eq. (2) of the main text. Similarly to our analysis in the main text, we impose a cap $\psi_{\max}$ on fitness, set to a finite value to simulate the scenario of a bounded fitness.

In Supplementary Fig. 3 we show the pandemic phase-diagrams under β-mutations for different values of $\psi_{\max}$. Similarly to the case of mutating α we find, again, the predicted phases: *infection-free*, *mutation-driven*, and *volatile* for finite $\psi_{\max}$ (Supplementary Fig. 3). Interestingly, we find that here the volatile phase emerges only at extreme values of σ, being *narrower* than that under α-mutations. Indeed, in Fig. 3 of the main text we enter the volatile phase already at $\sigma \sim 1$, being extremely volatile when $\sigma \sim 10$. Here, in contrast, we only observe the *tip* of the volatile phase at around $\sigma \sim 10$, as the mutated phase boundary gradually tends towards greater R_0.

To understand this difference consider the case of extreme mutations in α, *i.e.* large σ. Under such mutations, a relatively fit strain (small α) may reproduce in the next generation into an extremely unfit offspring with much larger α. Having such high recovery rate, the offspring population will be eliminated within a small short period, before having time to generate additional infections, thus terminating the highly fit evolutionary branch. This effect is less dramatic when the evolution impacts β. Now, indeed, a highly fit strain (large β) can still give rise to unfit offspring (small β), yet these offspring will continue to reproduce for some time, as their α remains unchanged. Hence the effect of volatile mutations is more subtle

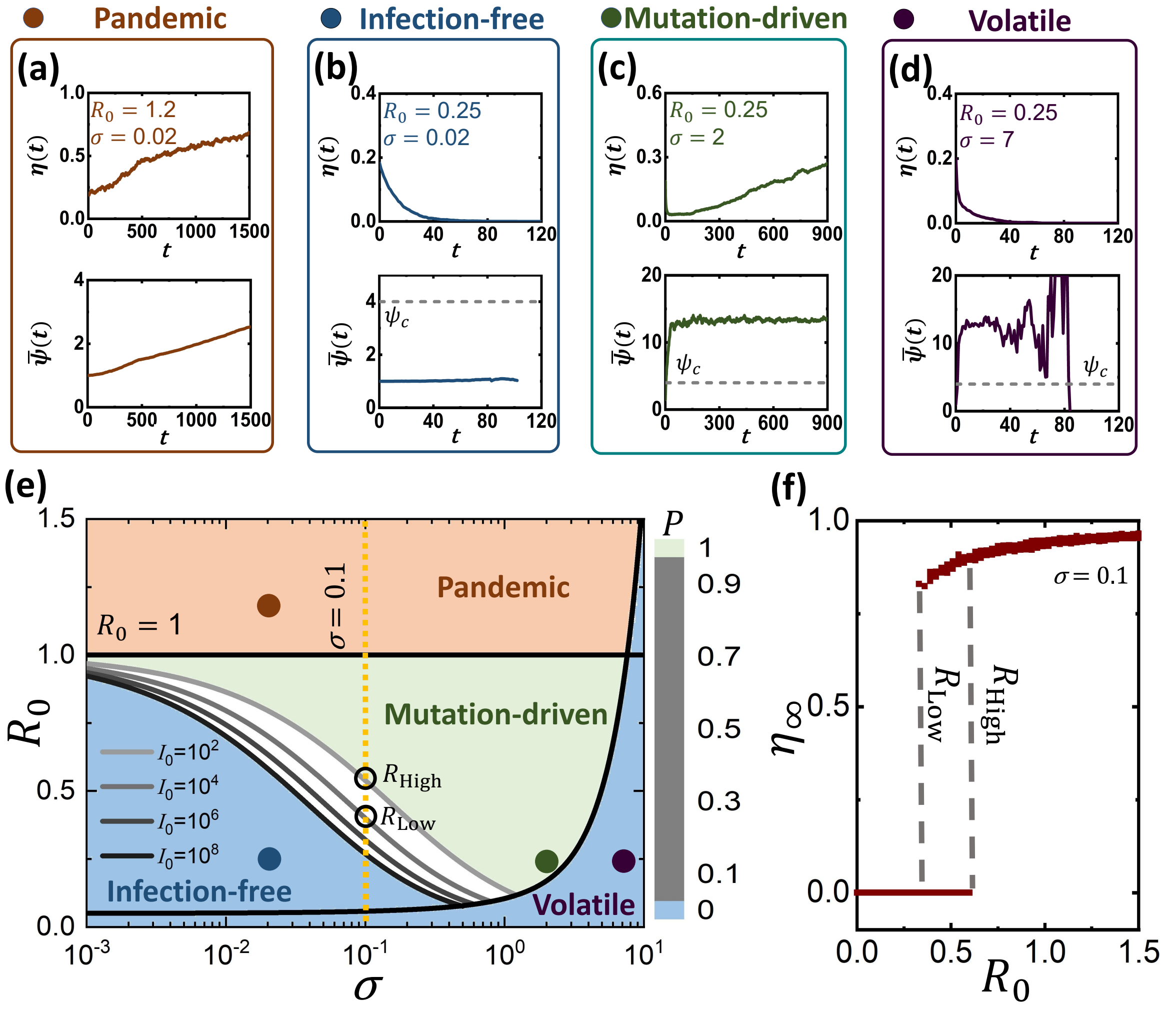

Supplementary Figure 2: Phase diagram under SIS dynamics. Similar to the case of SIR, we observe the four phases: (a) pandemic; (b) infection-free; (c) mutation-driven; (d) volatile. (e) The σ, R_0 phase diagram. The left boundary depends on $\mathcal{I}_0$ (grey dashed lines) and hence, for a given σ the transition is at R_{High} if we approach from the infection-free state, and R_{Low} in case we start from an already pandemic pathogen. (f) The result is a hysteresis phenomenon: starting from $R_0 = 0$, the pathogen only spreads when $R_0 \geq R_{\text{High}}$, yet if we wish to reverse the transition, we must reach $R_0 \leq R_{\text{Low}}$.

under β-mutations.

4 Correlated inter/intra-host fitness

So far, we discussed the case where the intra-host and inter-host fitness parameters $\varphi_\mu(i)$ and ψ_μ are assigned independently. This lead to the observed zero-mean random walk in the inter-host fitness space, resulting in the Gaussian form of $f_t(\psi)$ in (1.4). More generally, some phenotypical traits can benefit both the intra and the inter-host fitness, introducing a potential correlation between $\varphi_\mu(i)$ and ψ_μ. The result is a biased random walk in which the observed fitness gains follow

$$\Delta\psi \sim \mathcal{N}(\chi, \sigma^2) \tag{4.1}$$

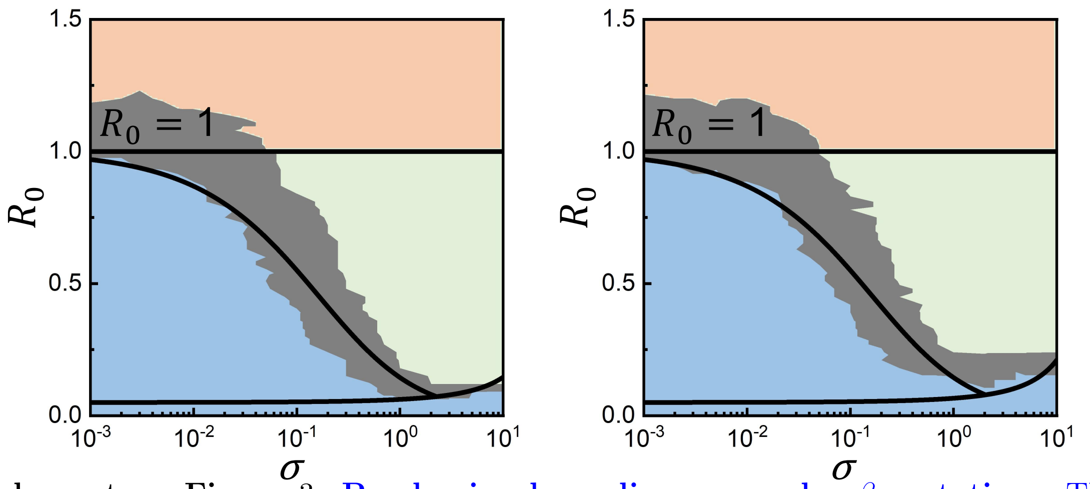

Supplementary Figure 3: **Pandemic phase-diagram under β-mutations**. The the phase diagram for SIR dynamics under $\psi_{\max} = 20$ (left) and $\psi_{\max} = 10$. The theoretical predictions as in the main text are also shown (black solid lines).

with χ being positive if $\varphi_\mu(i)$ and ψ_μ are positively correlated, and negative if the opposite is the case. Hence, depending on χ the intra-host selection either aids the inter-host transmissibility or vice versa; our main text discussion, *i.e.* Eq. (10), focuses on the specific case of $\chi = 0$. In Supplementary Figs. 4 and 5 we reconstruct the pandemic phase-diagrams for different values of χ. As expected, the mutation-driven phase is broadened in case of positive χ, where intra-host selection contributes to the inter-host fitness, and narrowed under negative χ, where the two fitness parameters conflict.

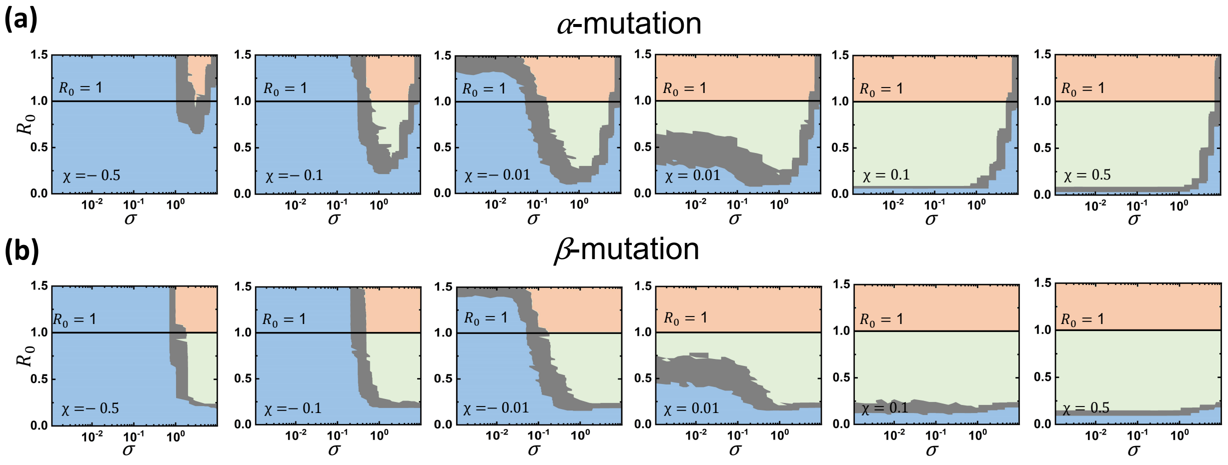

Supplementary Figure 4: **Correlated inter/intra-host SIR evolutionary dynamics**. (a) The σ, R_0 phase-diagrams under α-mutations, for an array of χ values, from $\chi = -0.5$ to $\chi = 0.5$. As expected the mutation-driven phase (green) favors positive χ. (b) Similar results are obtained under β-mutations.

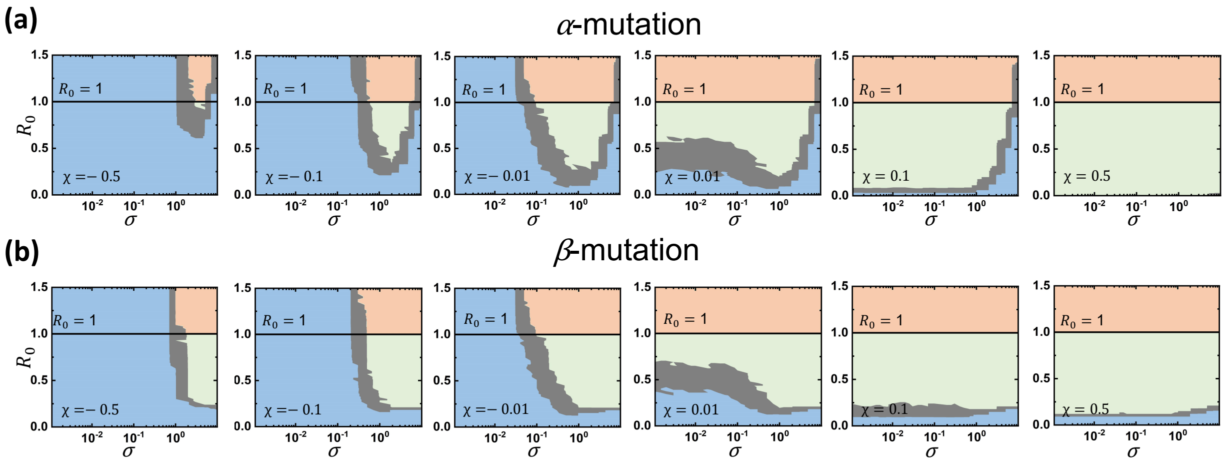

Supplementary Figure 5: **Correlated inter/intra-host SIS evolutionary dynamics.** We observe similar results to those obtained under SIR (Supplementary Fig. 4).

5 Numerical simulations and data analysis

5.1 Simulating SIR dynamics

To construct Figs. 2 - 4 of the main text we implemented the SIR model stochastically on a random network. The network was constructed using the Erdős-Rényi random graph model $G(N, p)$ with $N = 5,000$ nodes and connection probability $p = 1.5 \times 10^{-3}$, yielding an average degree of $\bar{k} = 15$. The dynamics were simulated via Gillespie with the transitions given by Eqs. (7) - (9) of the main text. The disease parameters were set to $\alpha_0 = 0.1$ and β_0 was varied as $\beta_0 = \alpha_0 R_0/\bar{k}$ to simulate the different values of R_0. In our simulations we instigated an outbreak by setting the initial condition to $\eta(0) = 0.02$.

Our phase-diagrams were obtained by varying σ and R_0 as specified in the figure captions. To evaluate whether the system is in the infection-free vs. pandemic phase we extracted the long term prevalence via r_∞, capturing the fraction of nodes in the recovered phase at $t \to \infty$. We consider the system to be at the pandemic state in case $r_\infty \geq 0.2$ after $4,000$ Gillespie iterations. Each of the $1,050$ R_0, σ data-points is a result of simulating 50 independent realizations, from which we extracted the probability P to observe a pandemic outcome.

The predicted phase boundaries in Eqs. (15) and (16) of the main text capture the functional form of the critical transition point, but not necessarily its precise value. Therefore we exploited a degree of freedom to multiply these predictions by a constant of order unity. Specifically, to obtain the black solid lines in Figs. 2i and 3d of the main text we multiplied Eq. (15) by 5.5, and Eq. (16) by 0.5. In Supplementary Fig. 3 we used the factors 5 for Eq. (15), and 1.5 and 2 for Eq. (16) in panels (a) and (b), respectively.

5.2 Extracting σ

The observed inter-host mutation rate σ arises from the detailed intra-host evolutionary dynamics. These internal dynamics are realized numerically through the following steps:

(i) **Fitness distribution**. We begin with a set of $\mu_{\max}$ potential strains and assign each with an intra-host fitness $\varphi_\mu(i) \sim \mathcal{N}(1, \sigma_\varphi^2)$ and an inter-host fitness $\psi_\mu \sim \mathcal{N}(1, \sigma_\psi^2)$, independently for all $\mu = 1, \dots, \mu_{\max}$. Hence, on average the fitness parameters are centered around $(1, 1)$, the wild-type fitness, but with some variability governed by $\sigma_\varphi, \sigma_\psi$. To avoid negative fitness we truncate both normal distributions at zero • In our simulation in Fig. 1e of the main text we set $\mu_{\max} = 200, \sigma_\varphi = 0.2$ and $\sigma_\psi = 0.2$.

(ii) **Initial pathogen pool**. We then initiate a population of n individual pathogens $Y = 1, \dots, n$, chosen at random from our pool of $\mu_{\max}$ potential strains. We denote pathogen Y's selected strain by y; hence the upper case Y signifies a specific copy of one of the n pathogens, while the lower-case $y = 1, \dots, \mu_{\max}$ indicates its strain type. For example, pathogen Y's fitness parameters are $\varphi_y(i), \psi_y$, the fitness values assigned to the strain $\mu = y$ • In Fig. 1e we used a population of $n = 2,000$ individual pathogens.

(iii) **Replication and selection**. We allow the population to evolve across ρ replication cycles. At every cycle, we run over all $Y = 1, \dots, n$ pathogens and remove pathogen Y with a constant probability $q_- = 0.2$. For every removed pathogen we allow another pathogen X to replicate, this time with a fitness dependent probability $q_+ \propto \varphi_x(i)$. Therefore, we remain with a constant pathogen population of size n, whose composition changes in favor of the strains with higher intra-host fitness $\varphi_x(i)$, as they reproduce with increased probability • The number of replications used in Fig. 1e was set to $\rho = 10^4$.

(iv) **Mutation**. When pathogen X of type x replicates it mutates with probability p to one of its adjacent strains types $x \pm 1$. With probability $1 - 2p$ its offspring remains of type x • We used the mutation probability $p = 0.02$.

Following ρ replication cycles we arrive at a population of n evolved strains, selected for higher intra-host fitness (Supplementary Fig. 6a). We perform 2×10^4 independent realizations, and from the statistics of this population extract the two distributions shown in Fig. 1e of the main text, $f_i(\varphi)$ and $f_i(\psi)$ - the latter providing us with the observed mutation rate σ.

Parameters affecting σ. Our intra-host dynamics are all encapsulated within a single relevant observable parameter σ. To examine how σ is impacted by these internal processes we test in Supplementary Fig. 6b-d the dependence of σ on (i) the number of replications ρ; (ii) the intra-host mutation probability p; (iii) the intra-host fitness variability across all strains σ_φ; (iv) the inter-host fitness variability σ_ψ.

We find that σ is primarily sensitive to the latter two, *i.e.* the intrinsic variability among the strains, but largely insensitive to ρ and p. To understand this, consider the limits $\rho \to \infty, \sigma_\varphi \to 0$ and $p \to 1$. Under these conditions, intra-host selection will play no role, since all strains have similar intra-host fitness, and the original pathogen pool will spread out almost evenly across all $\mu_{\max}$ strains, in effect, *forgetting* its initial condition. As a result $f_i(\psi)$ will be driven by the intrinsic variability in the inter-host fitness of the strains, *i.e.* $\sigma \approx \sigma_\psi$. Therefore, adding more replication cycles (ρ) or mutation instances (p) has a limited effect, as - at most - the population reaches a uniform coverage of all strains - a point in which fitness variability is only limited by how different the strains are among themselves (σ_ψ).

Next, we consider the impact of σ_φ. In case this parameter is large, the strains have a broadly distributed intra-host fitness, resulting in strong selection pressure towards the fittest strains. After sufficient replication/selection this will lead to a narrow spectrum of surviving strains - those with the highest $\varphi_\mu(i)$. As a consequence the final composition of the intra-host pathogen population $\mathcal{Z}_i$ will exhibit small variability, concentrated primarily on this small sample of fit strains, and hence a relatively narrow $f_i(\psi)$. Indeed, as Supplementary Fig. 6d indicates, σ decreases with σ_φ.

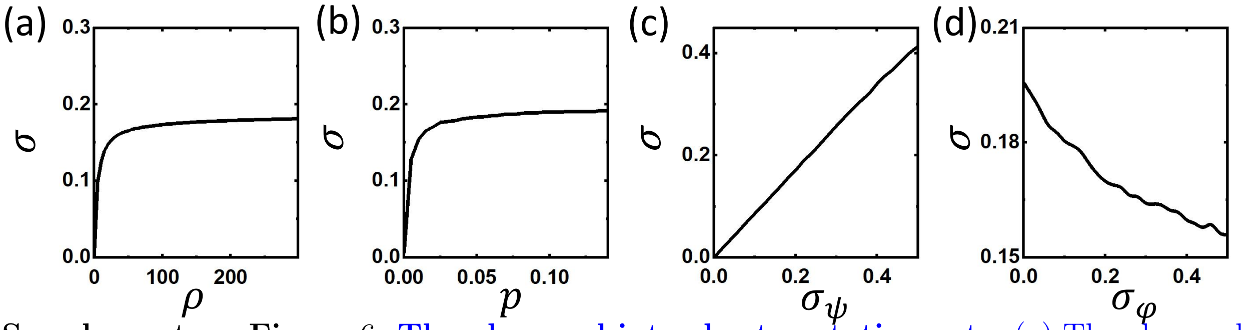

Supplementary Figure 6: The observed inter-host mutation rate. (a) The observed inter-host mutation rate σ of the pathogens vs. the number of replication cycles ρ. (b) The observed σ vs. the intra-host mutation probability p. (c) σ vs. σ_ψ and (d) σ vs. σ_φ.

5.3 Simulating SARS-CoV-2 transmission

To simulate the spread of COVID-19 we used an elaborate compartmental model, taking all transitions and rates from empirical data on SARS-CoV-2[11], as detailed in Fig. 5a of the main text. The cycle includes the following set of transitions:

$$
\begin{aligned}
&S + I \xrightarrow{\beta_0} E, && E \xrightarrow{1/4,30\%} I_{\mathrm{AS}} \\
&E \xrightarrow{1/3,70\%} I_{\mathrm{PS}}, && I_{\mathrm{AS}} \xrightarrow{1/7} R \\
&I_{\mathrm{PS}} \xrightarrow{1/2,55\%} I_{\mathrm{M}}, && I_{\mathrm{PS}} \xrightarrow{1/2,10\%} I_{\mathrm{S}} \\
&I_{\mathrm{PS}} \xrightarrow{1/2,5\%} I_{\mathrm{C}}, && I_{\mathrm{M}} \xrightarrow{1/5} R \\
&I_{\mathrm{S}} \xrightarrow{1/15,85\%} R, && I_{\mathrm{S}} \xrightarrow{1/15,15\%} D \\
&I_{\mathrm{C}} \xrightarrow{1/16,50\%} R, && I_{\mathrm{C}} \xrightarrow{1/16,50\%} D,
\end{aligned}
\tag{5.1}
$$

where on top of each arrow we specify the transition rate (days^{-1}) and branching probability. For example, exposed individuals (E) transitions to the asymptomatic state (I_{AS}), with probability 30% after an average of 4 days, and hence the transition rate is 1/4 days^{-1}. In (5.1) I represents the infectious population, *i.e.* $I_{PS} \cup I_{AS}$, both of whom contribute to the spread. The remaining infected groups (I_M, I_S, I_C) are isolated and hence cannot infect others.

With every infection the virus evolves, shifting across different strains μ, each with its cross-infection fitness ϕ_μ. This is captured by a Gaussian random walk along in $z = \mu/\mu_{\max}$,

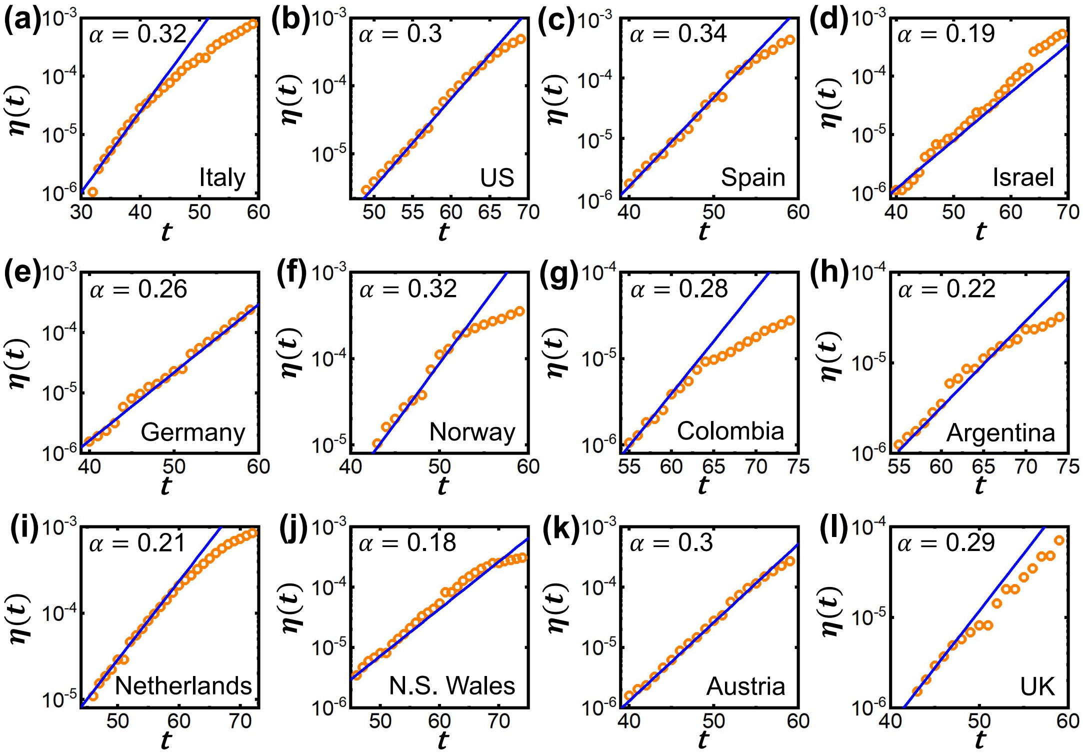

Supplementary Figure 7: **Estimating β_0 for SARS-CoV-2**. The prevalence $\eta(t)$ vs. t in 12 selected countries as obtained from empirical data on symptomatic carriers of SARS-CoV-2 (orange circles). The early stages of the spread can be well approximated by an exponential growth of the form $\eta(t) \sim e^{at}$ (blue solid lines), with an average growth rate of $a = 0.27$ days^{-1}. As the exponential growth typically continues for a period of several days posterior to the instigation of the mitigation policy, we used only the data up to 3 days after the implementation of social distancing to estimate β_0.

starting from $z = 0$ and randomly shifting along the positive z-axis. To enable cross-infection we add to (5.1) the additional transition

$$I_i + R_j \xrightarrow{\gamma_\mu} I_i + E_j, \tag{5.2}$$

in which an individual i transmits a variant μ to a recovered individual j at a rate γ_μ. The rate of this transition is

$$\gamma_\mu = \phi_\mu \beta_0, \tag{5.3}$$

namely the probability to reinfect (ϕ_μ) times the original SARS-CoV-2 infection rate (β_0). Hence, here the evolutionary dynamics does not affect the infection rate, which remains β_0, just the reinfection probability ϕ_μ, which is zero for the wild-type, and potentially higher for the evolved strains $\mu > 0$. Here, we set ϕ_μ to

$$\phi_\mu = \frac{z^h}{z_r^h + z^h}, \tag{5.4}$$

tending to zero for small z (genetically similar to wild-type), and approaching unity for large

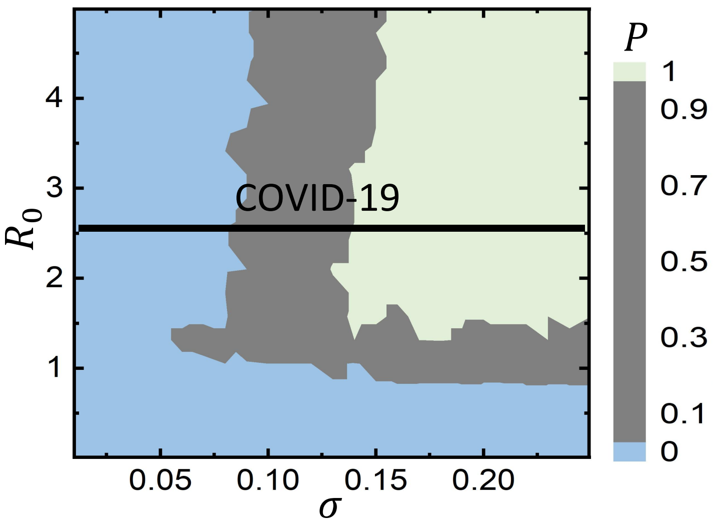

Supplementary Figure 8: **COVID-19 reemergence**. The probability P to observe a breakthrough mutation leading to a second wave of infections in function of σ, R_0, under the COVID-19 disease cycle. Here we assume that only 70% of mild symptomatic individuals isolate.

z (highly evolved compared to wild-type). Taken together, the virus spreads via (5.1), and at the same time performs a random walk in z. This, in turn, translates to an evolutionary path in ϕ-space, gaining ($\Delta z > 0$) or losing ($\Delta z < 0$) reinfection fitness. In our simulations we set $z_r = 0.3$ and $h = 10$.

The COVID-19 cycle was implemented on the Erdős-Rényi network described above, from $t = 0$ to 200 days at 15 minute resolution, *i.e.* 96 time-stamps per day. The initial condition was set to $\eta(0) = 2 \times 10^{-3}$, a 0.2 percent of *exposed* individuals. In (5.1) we assume symptomatic individuals are isolated, and hence the spread is mediated by the pre-symptomatic (PS state) and by the asymptomatic (AS) infected individuals. This leaves an average infection window of 2 days for the symptomatic carriers (70%) and 7 days for the asymptomatic (30%). Hence, on average the time window for individuals to propagate the viruse is $0.3 \times 7 + 0.7 \times 2 = 3.5$ days. This helps us evaluate the effective recovery rate as $\alpha_0 = 1/3.5 = 0.29$ days^{-1}.

While all transition rates/probabilities in (5.1) are available from data on the SARS-CoV-2 disease timescales, the infection rate β_0 is unknown, and must be extracted from the observed spreading dynamics. To achieve this we collected empirical data on the early stages of the spread, prior to the imposition of mitigation policies (Supplementary Fig. 7). The data indicate that the initial rise in the prevalence of SARS-CoV-2 exhibits an exponential growth of the form $\sim e^{at}$, with $a \approx 0.27$ days^{-1}. We therefore seek the best fit infection rate β_0, such that in our simulated spread, we have

$$\mathcal{I}(t) \sim e^{0.27t}, \tag{5.5}$$

where $\mathcal{I}(t) = I_M(t) + I_S(t) + I_C(t)$, *i.e.* all symptomatic individuals. We find that the best fit, under our simulated conditions, is observed for $\beta_0 \approx 0.05$ days^{-1}. Using $R_0 = \beta_0 \overline{k}/\alpha_0$, and taking $\alpha_0 = 0.29$, this corresponds to $R_0 \approx 2.6$, as stated in the main text. In our

analysis, here in Supplementary Fig. 8 and in Fig. 5 of the main-text, we examined a range of pandemic scenarios, from $R_0 = 0$ to $R_0 = 5$. This covers our extracted $R_0 = 2.6$ together with quite significant margins that extend well beyond our actual uncertainty pertaining to the COVID-19 parameters.

Non-compliance. To complete our analysis we also consider the case where some of the symptomatic individuals remain active, as indeed, not all of the population abides by the isolation requirements. We, therefore allow 30% of the mild-symptomatic individuals (I_M) to transmit the virus, adding them to the I group, while the remaining 70% isolate as instructed. As Supplementary Fig. 8 indicates, this has little impact on the resulting phase-diagram, which continues to follow the same patterns as the main text Fig. 5f.

Supplementary references

[1] Pastor-Satorras, R., Castellano, C., Van Mieghem, P. and Vespignani, A., Epidemic processes in complex networks. Reviews of modern physics, 87, 925 (2015).

[2] Gómez-Gardeñes, J., Lotero, L., Taraskin, S. N. and Pérez-Reche, F. J., Explosive contagion in networks, Sci. Rep. 6, 19767 (2016).

[3] Liu, Q., Wang, W., Tang, M., Zhou, T. and Lai, Y., Explosive spreading on complex networks: The role of synergy, Phys. Rev. E 95, 042320 (2017).

[4] Wu, J., Zheng, M., Xu, K. and Gu, C., Effects of two channels on explosive information spreading, Nonlinear Dynamics, 99, 2387-2397, (2020).

[5] Hébert-Dufresne, L., Scarpino, S. V. and Young, J., Macroscopic patterns of interacting contagions are indistinguishable from social reinforcement, Nature Physics 16, 426 (2020).

[6] Gross, T., Lima, C. J. D. and Blasius, B., Epidemic dynamics on an adaptive network, Phys. Rev. Lett. 96, 208701 (2006).

[7] Böttcher, L., Woolley-Meza, O., Goles, E., Helbing, D. and Herrmann, H. J., Connectivity disruption sparks explosive epidemic spreading, Phys. Rev. E 93, 042315 (2016).

[8] Zhang, X., Ruan, Z., Zheng, M., Barzel, B. and Boccaletti, S., Epidemic spreading under infection-reduced-recovery, Chaos, Solitons and Fractals 140, 110130 (2020).

[9] Boccaletti, S. et al. Explosive transitions in complex networks? structure and dynamics: Percolation and synchronization, Physics Reports 660, 1-94 (2016).

[10] D'Souza, R. M., Gómez-Gardeñes, J., Nagler, J. and Arenas, A., Explosive phenomena in complex networks[J]. Advances in Physics, 2019, 68(3): 123-223.

[11] D. Meidan, N. Schulmann, R. Cohen, S. Haber, E. Yaniv, R. Sarid and B. Barzel, Alternating quarantine for sustainable epidemic mitigation, Nature Communications, 12, 1-12 (2021).
